# Supplementary material for: Acceleration of U.S. Southeast and Gulf coast sea-level rise amplified by internal climate variability
Source: Nat Commun. 2023 Apr 10;14:1935. doi: 10.1038/s41467-023-37649-9 (PMC10086026; doi:10.1038/s41467-023-37649-9)
Supplement: Supplementary file 1 — Supplementary Information [file 41467_2023_37649_MOESM1_ESM.pdf]

Supplementary Material to:

Acceleration of U.S. Southeast and Gulf Coast Sea-Level Rise Amplified by Internal Climate Variability

Sönke Dangendorf[1], Noah Hendricks[2], Qiang Sun[1], John Klinck[2], Tal Ezer[2], Thomas Frederikse[3], Francisco M. Calafat[4], Thomas Wahl[5], and Torbjörn E. Törnqvist[6]

[1]Department of River-Coastal Science and Engineering, Tulane University, 6823 St. Charles Avenue New Orleans, LA 70118, USA, Email: sdangendorf1@tulane.edu

[2]Center for Coastal Physical Oceanography, Department of Ocean and Earth Sciences, Old Dominion University, 4111 Monarch Way, Norfolk, 23508, USA

[3]Jet Propulsion Laboratory, California Institute of Technology, Pasadena, CA, USA

[4]National Oceanography Centre, 6 Brownlow Street, Liverpool, L3 5DA, UK

[5]Department of Civil, Environmental and Construction Engineering, University of Central Florida, Orlando, 32816, Florida, USA

[6]Department of Earth and Environmental Sciences, Tulane University, 6823 St. Charles Avenue New Orleans, LA 70118, USA

Table of Content

Supplementary Fig. 1 | Study area and tide gauge locations	3
Supplementary Fig. 2 | Observed linear mean sea level (MSL) trends from tide gauges along the North American East and Gulf Coasts, 1900-2021	4
Supplementary Fig. 3 | Observed rates of mean sea level (MSL) change and different contributing processes averaged along the North American East and Gulf Coasts over the period 1900 to 2021	5
Supplementary Fig. 4 | Coherence of nonlinear sterodynamic sea level (SDSL) rates	6
Supplementary Fig. 5 | Latitudinal dependence of sterodynamic sea level (SDSL) and wind driven Rossby waves in the North Atlantic Ocean	
Supplementary Fig. 6 | Spatial Correlations between Caribbean Sea Level and the larger northwestern Atlantic region	7
Supplementary Fig. 7 | Principal components and spatial patterns of Rossby waves west of 50°W	8
Supplementary Fig. 8 | Nonlinear vertical land motion (VLM) in the Gulf of Mexico	9
Supplementary Fig. 9 | Comparison between linear relative mean sea level (MSL) and vertical land motion (VLM) rates over the period 1900 to 2021	10
Supplementary Fig. 10 | Correlation between Rossby waves and sea surface height from satellite altimetry	11
Supplementary Tab. 1 | List of all tide gauge records used in this study	12
Supplementary Tab. 2 | List of all climate models used in this study	13

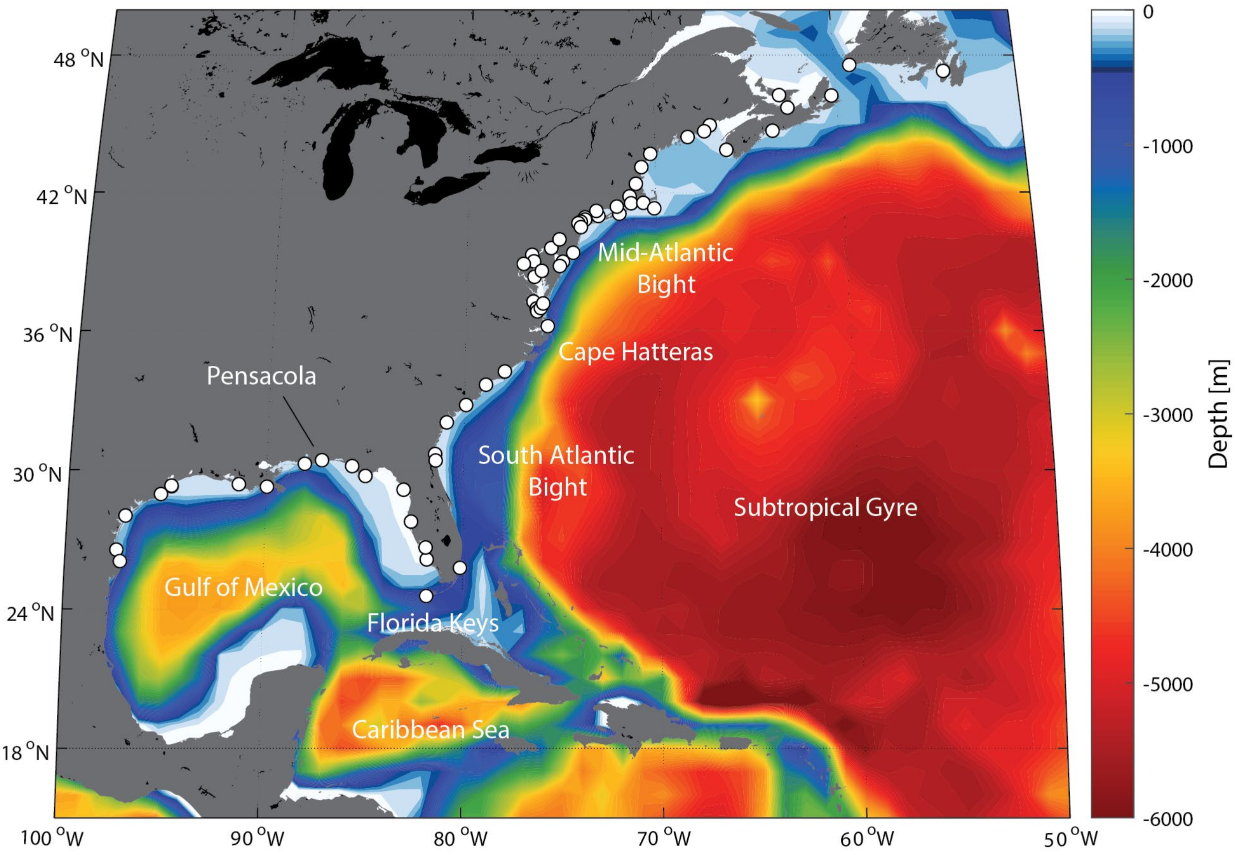

Supplementary Fig. 1 | Study area and tide gauge locations. Contours represent the ocean bathymetry. Tide gauge locations are marked by the white circles.

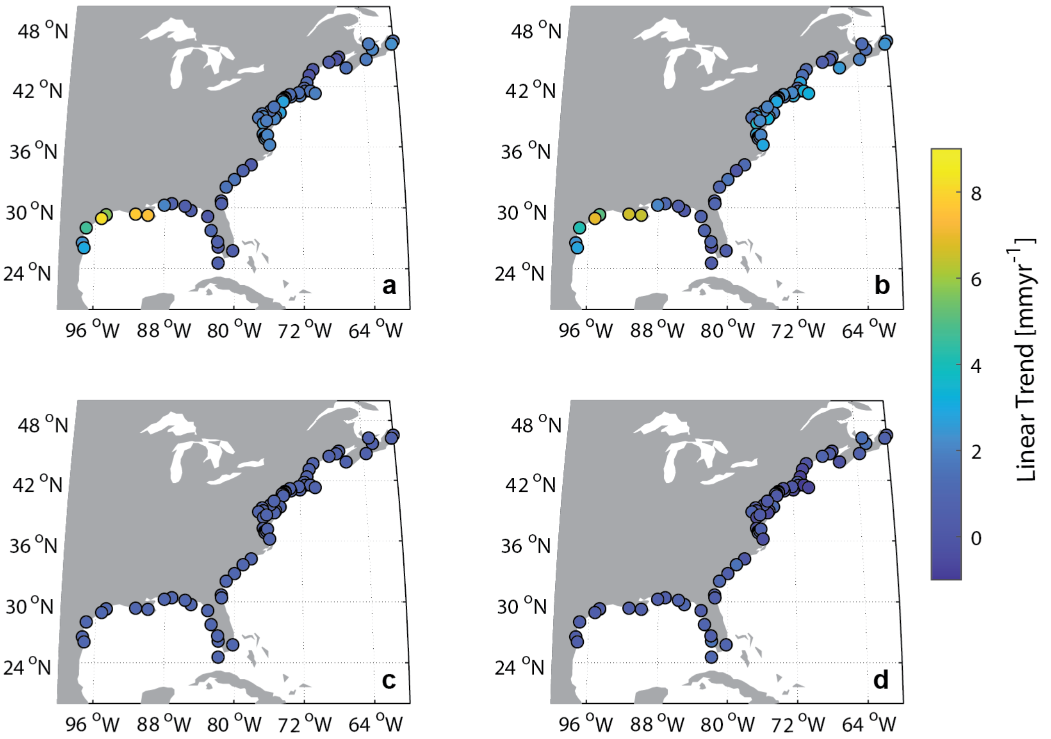

Supplementary Fig. 2 | Observed linear mean sea level (MSL) trends from tide gauges along the North American East and Gulf Coasts, 1900-2021. Shown are the rates for **a** relative MSL, **b** vertical land motion (VLM), **c** effects of gravitation, rotation, and deformation (GRD) related to barystatic sea level change, and **d** sterodynamic sea level (SDSL). The individual contributions have been derived from sources described in the **Methods** section. The figure demonstrates, in correspondence to ref. 14, that VLM is the major source of spatial variability in the region. Note that linear trends have been calculated based on gap-filled records (see **Methods**).

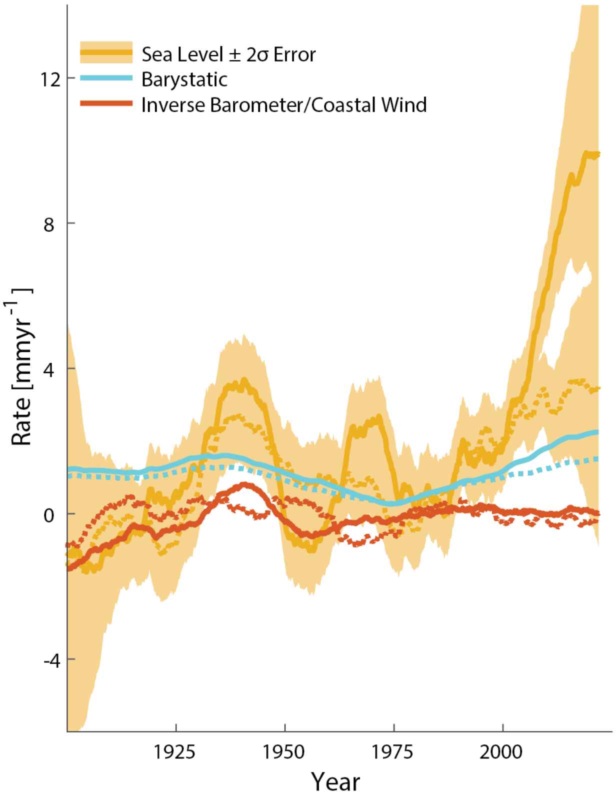

Supplementary Fig. 3 | Observed rates of mean sea level (MSL) change and different contributing processes averaged along the North American East and Gulf Coasts over the period 1900 to 2021. The results are shown for regional medians over all tide gauge records north (dotted lines) and south (solid lines) of Cape Hatteras after correction for vertical land motion. Rates have been derived from singular spectrum analysis (SSA) with a cutoff period of 30 years. Shadings represent the 2σ uncertainties of the nonlinear trends. Rates of the effects of gravitation, rotation, and deformation due to barystatic sea-level change are from ref. 7. Coastal wind plus inverted barometer contributions stem from ref. 20.

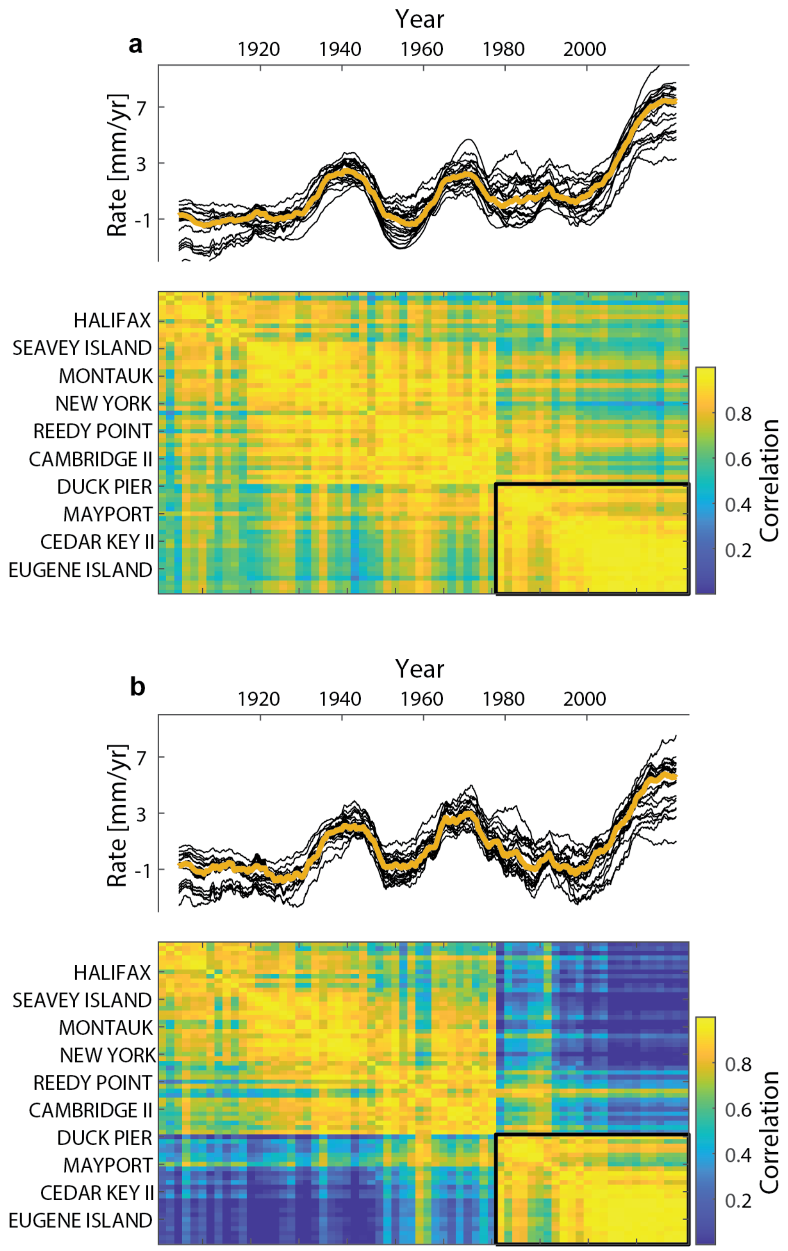

Supplementary Fig. 4 | Coherence of nonlinear sterodynamic sea level (SDSL) rates. Shown are the nonlinear trend rates of the SDSL residual (that is: tide gauge records minus effects of gravitation, rotation, and deformation due to barystatic sea level changes, vertical land motion, and inverse barometer effect) for all tide gauges south of Cape Hatteras (top panels) as well as their site-by-site correlation matrix along the entire coastline (ordered from north to southwest; lower panels) for (**a**) raw SDSL, and (**b**) SDSL rates corrected for the forced response from model of the Coupled Model Intercomparison Project 6 (CMIP6).

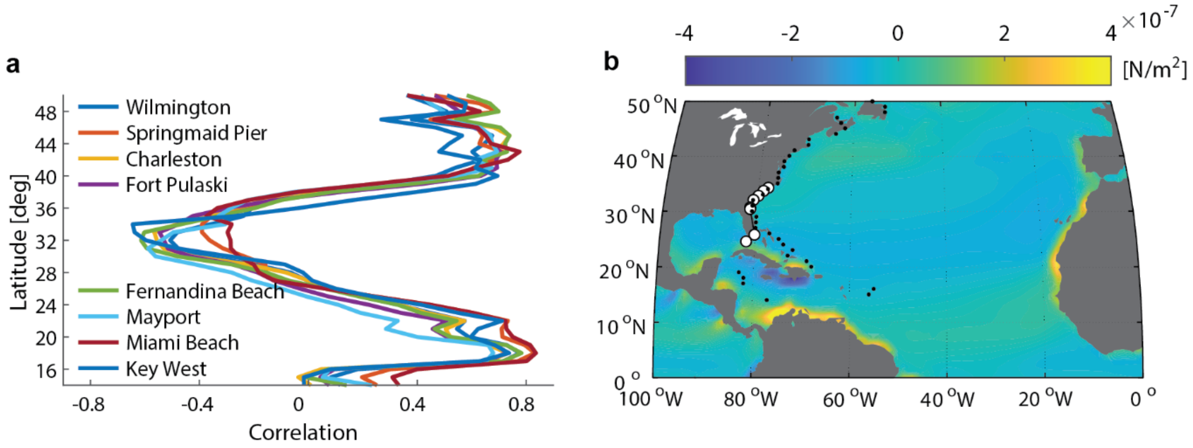

Supplementary Fig. 5 | Latitudinal dependence of sterodynamic sea level (SDSL) and wind driven Rossby waves in the North Atlantic Ocean. a Correlation between coastal SDSL variability at individual U.S. Southeast coast locations (white circles in **b**) and outputs from the 1.5-layer reduced gravity model as a function of latitude (calculated over 1940 to 2015). **b** Average wind stress curl climatology over the most recent 30-year period from 1986 to 2015. Black little dots show the western boundary to which wind stress curl has been integrated with 1.5-layer reduced gravity model for the correlation plot in **a**.

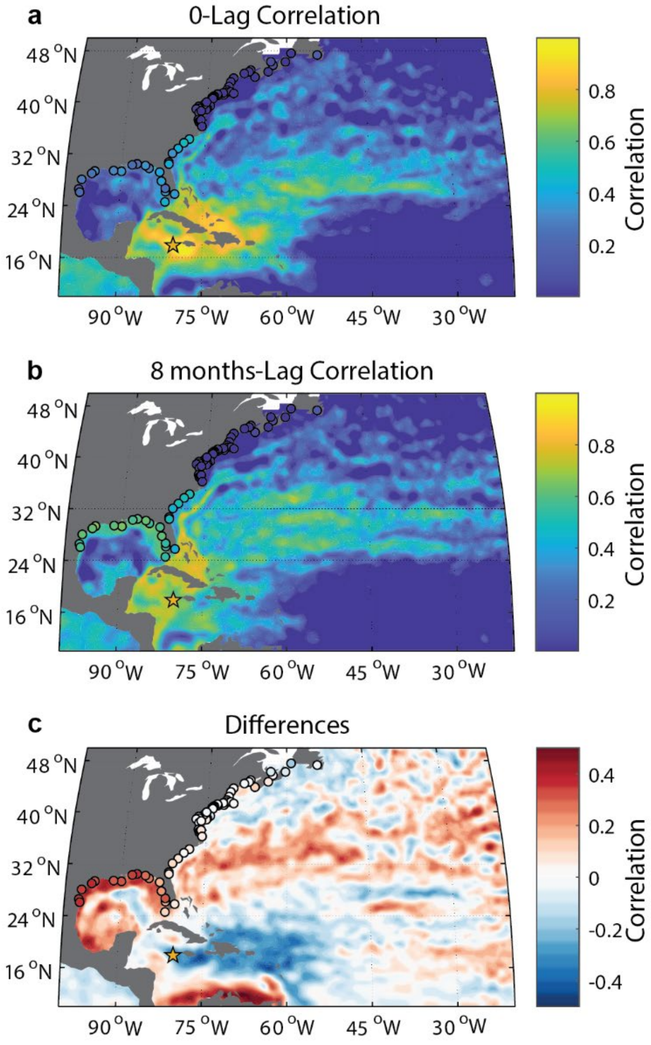

Supplementary Fig. 6 | Spatial Correlations between Caribbean Sea Level and the larger northwestern Atlantic region. Correlations between a central point in the Caribbean Sea (orange pentagram) and each other grid point from satellite altimetry over the period 1993 to 2019. Correlation maps are shown for a zero (**a**) and an eight-months (**b**) lag. **c** Differences between the two maps in **a** and **b**. The circles show the corresponding correlations with individual tide gauge records. All time series have been smoothed by a moving average filter with a cutoff period corresponding to 12 months.

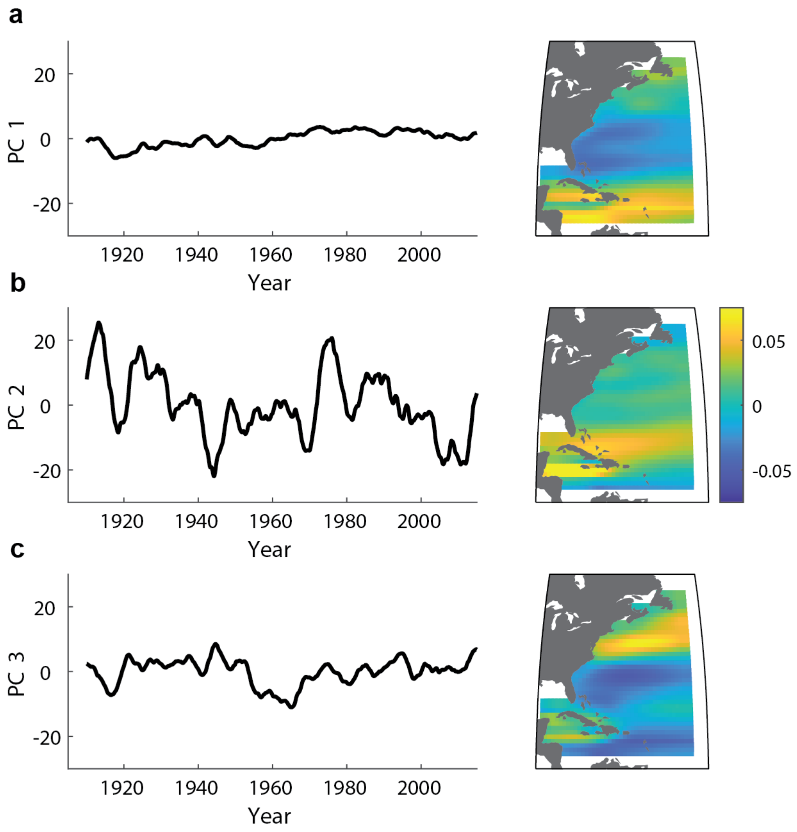

Supplementary Fig. 7 | Principal components and spatial patterns of Rossby waves west of 50°W. a Principal components of Rossby waves west of 50°W. Amplitudes reflect their total contribution to coastal sterodynamic sea level (SDSL) as derived from stepwise regression (see **Methods**). The stepwise regression selected principal components 1, 2, and 3 to be finally used. **b** The corresponding spatial patterns.

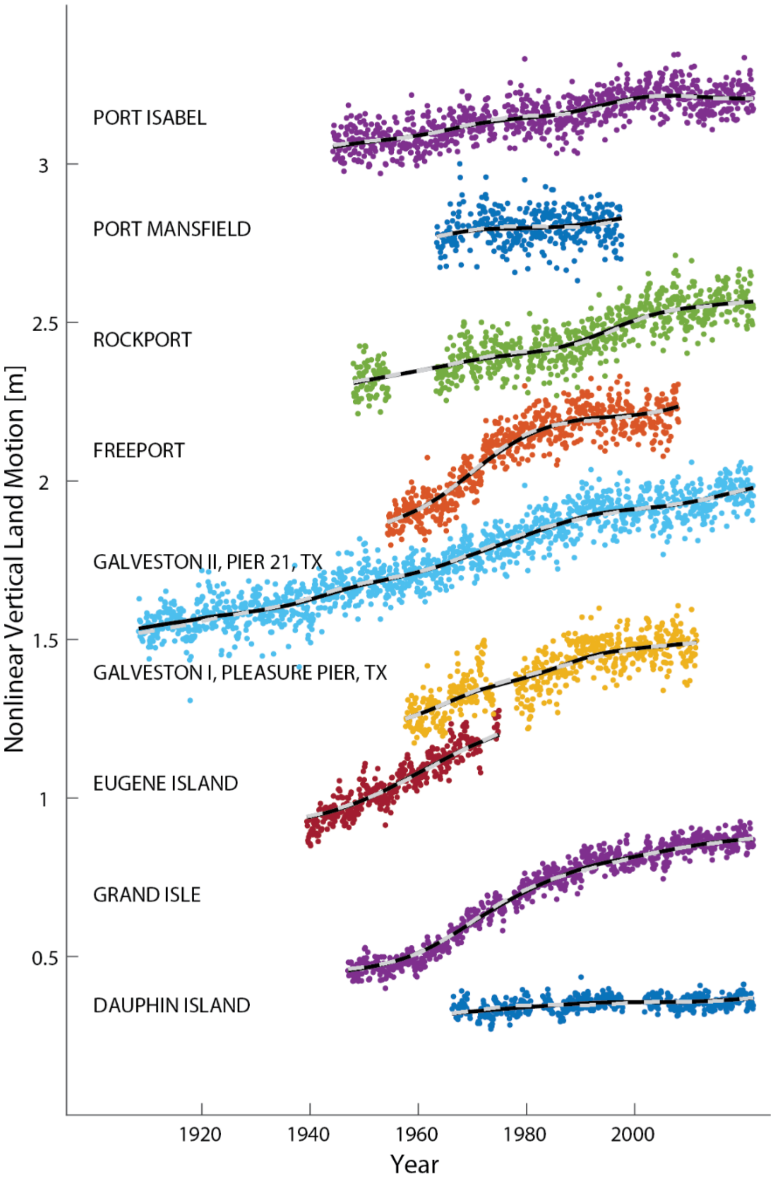

Supplementary Fig. 8 | Nonlinear vertical land motion (VLM) in the Gulf of Mexico. Shown are the residuals (colored dots) between tide gauge records in the western Gulf of Mexico and Pensacola, Florida. The tide gauge records have been corrected for effects of gravitation, rotation, and deformation due to barystatic sea level changes, Glacial Isostatic Adjustment, the inverted barometer effect, and coastal winds before calculating the differences to Pensacola. The corresponding VLM changes, inferred from a singular spectrum analysis (SSA) with an embedding dimension corresponding to a cutoff period of 30 years (see **Methods**), are shown as thick black lines. As validation the same VLM changes inferred from the differences to the record of Cedar Key, Florida, are also shown (grey dashed line). Both agree very well, indicating that the resulting nonlinear VLM estimates are robust.

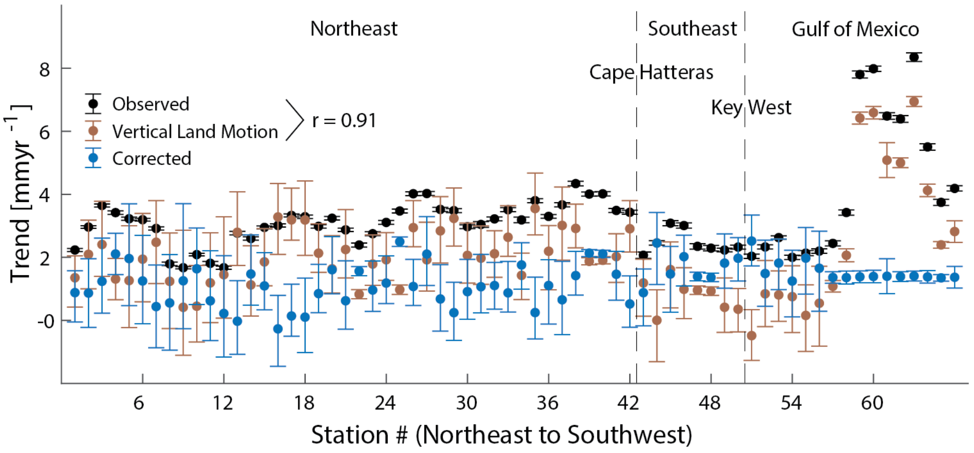

Supplementary Fig. 9 | Comparison between linear relative mean sea level (MSL) and vertical land motion (VLM) rates over the period 1900 to 2021. Shown are the linear trends (dots) with their 2σ standard error (bars). The dotted lines represent geographical borders at Cape Hatteras and Key West.

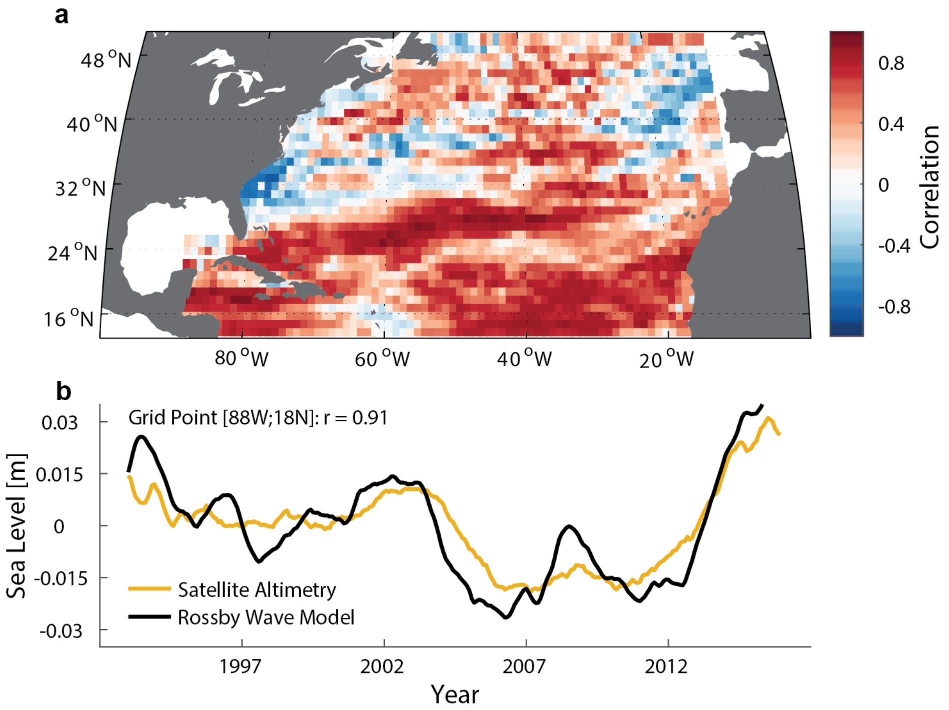

Supplementary Fig. 10 | Correlation between Rossby waves and sea surface height from satellite altimetry. a Pointwise correlation between the outputs from the 1.5-layer reduced gravity model and satellite altimetry at each location over the North Atlantic over their overlapping period from 1993 to 2015. Both time series have been linearly detrended and smoothed with a moving average filter with a cutoff period of 48 months. **b** Detrended and smoothed anomalies at an individual grid point at 88°W and 18°N.

Supplementary Tab. 1 | List of all tide gauge records used in this study. Shown are tide gauge name, location, and coverage.

Station Name	Location		Coverage [%]
	Lon [°]	Lat [°]	
'ARGENTIA'	-53.98	47.30	37.16
'PORT AUX BASQUES'	-59.13	47.57	45.77
'NORTH SYDNEY'	-60.25	46.22	40.51
'PICTOU'	-62.70	45.68	22.27
'CHARLOTTETOWN'	-63.12	46.23	73.70
'HALIFAX'	-63.58	44.67	75.61
'YARMOUTH'	-66.13	43.83	41.53
'EASTPORT'	-66.98	44.90	71.04
'CUTLER II'	-67.30	44.64	21.04
'BAR HARBOR, FRENCHMAN BAY, ME'	-68.21	44.39	54.85
'PORTLAND (MAINE)'	-70.25	43.66	89.96
'SEAVEY ISLAND'	-70.74	43.08	43.58
'BOSTON'	-71.05	42.35	82.04
'PROVIDENCE (STATE PIER)'	-71.40	41.81	58.67
'NEWPORT'	-71.33	41.51	74.04
'WOODS HOLE (OCEAN. INST.)'	-70.67	41.52	69.54
'NANTUCKET ISLAND'	-70.10	41.29	45.63
'MONTAUK'	-71.96	41.05	56.15
'NEW LONDON'	-72.09	41.36	66.12
'PORT JEFFERSON'	-73.08	40.95	26.50
'BRIDGEPORT'	-73.18	41.17	45.56
'NEW ROCHELLE'	-73.78	40.89	18.78
'WILLETS POINT'	-73.78	40.79	55.46
'NEW YORK (THE BATTERY)'	-74.01	40.70	99.32
'BERGEN POINT, STATEN IS.'	-74.14	40.64	26.02
'SANDY HOOK'	-74.01	40.47	71.38
'ATLANTIC CITY'	-74.42	39.36	84.49
'CAPE MAY'	-74.96	38.97	42.28
'LEWES (BREAKWATER HARBOR)'	-75.12	38.78	64.82
'REEDY POINT'	-75.57	39.56	28.28
'PHILADELPHIA (PIER 9N)'	-75.14	39.93	97.13
'BALTIMORE'	-76.58	39.27	97.75
'ANNAPOLIS (NAVAL ACADEMY)'	-76.48	38.98	73.77
'WASHINGTON DC'	-77.02	38.87	73.36
'SOLOMON''S ISLAND (BIOL. LAB.)'	-76.45	38.32	66.39
'CAMBRIDGE II'	-76.07	38.57	40.85
'GLOUCESTER POINT'	-76.50	37.25	40.30
'SEWELLS POINT, HAMPTON ROADS'	-76.33	36.95	77.25
'PORTSMOUTH (NORFOLK NAVY YARD)'	-76.29	36.82	42.55
'CHESAPEAKE BAY BR. TUN.'	-76.11	36.97	26.64
'KIPTOPEKE BEACH'	-75.99	37.17	57.24
'DUCK PIER OUTSIDE'	-75.75	36.18	29.44
'WILMINGTON'	-77.95	34.23	70.01
'SPRINGMAID PIER'	-78.92	33.66	29.85
'CHARLESTON I'	-79.93	32.78	82.17
'FORT PULASKI'	-80.90	32.03	70.49
'FERNANDINA BEACH'	-81.47	30.67	86.13
'MAYPORT'	-81.43	30.39	59.36
'MIAMI BEACH'	-80.13	25.77	38.25
'KEY WEST'	-81.81	24.56	88.66
'NAPLES'	-81.81	26.13	45.15
'FORT MYERS'	-81.87	26.65	43.37
'ST. PETERSBURG'	-82.63	27.76	61.41
'CEDAR KEY II'	-83.03	29.14	65.03
'APALACHICOLA'	-84.98	29.73	42.42
'PANAMA CITY, ST.ANDREWS BAY, FL'	-85.67	30.15	29.17
'PENSACOLA'	-87.21	30.40	79.78
'DAUPHIN ISLAND'	-88.08	30.25	36.75
'GRAND ISLE'	-89.96	29.26	58.95
'EUGENE ISLAND'	-91.39	29.37	27.19
'GALVESTON I, PLEASURE PIER, TX'	-94.79	29.29	40.30
'GALVESTON II, PIER 21, TX'	-94.79	29.31	92.69
'FREEPORT'	-95.31	28.95	43.72
'ROCKPORT'	-97.05	28.02	50.96
'PORT MANSFIELD'	-97.42	26.55	25.75
'PORT ISABEL'	-97.22	26.06	61.48

Supplementary Tab. 2 | List of all climate models used in this study. Shown all models distinguished after Climate Model Intercomparison Project (CMIP) groups 5 and 6. Also given are the forcings that have been used after 2005 to extend the historical model runs.

	Model	Forcing after 2005
CMIP5	'ACCESS1-0'	RCP 8.5
	'ACCESS1-3'	RCP 8.5
	'CMCC-CESM'	RCP 8.5
	'CMCC-CM'	RCP 8.5
	'CMCC-CMS'	RCP 8.5
	'CNRM-CM5'	RCP 8.5
	'CanESM2'	RCP 8.5
	'GFDL-CM3'	RCP 8.5
	'GFDL-ESM2G'	RCP 8.5
	'GFDL-ESM2M'	RCP 8.5
	'IPSL-CM5A-LR'	RCP 8.5
	'IPSL-CM5A-MR'	RCP 8.5
	'MPI-ESM-LR'	RCP 8.5
	'MPI-ESM-MR'	RCP 8.5
	'NorESM1-M'	RCP 8.5
	'NorESM1-ME'	RCP 8.5
CMIP6	'ACCESS-CM2'	SSP 585
	'ACCESS-ESM1-5'	SSP 585
	'CMCC-CM2-SR5'	SSP 585
	'CNRM-CM6-1-HR'	SSP 585
	'CNRM-ESM2-1'	SSP 585
	'CanESM5'	SSP 585
	'EC-Earth3'	SSP 585
	'GISS-E2-1-G'	SSP 585
	'IPSL-CM6A-LR'	SSP 585
	'MIROC6'	SSP 585
	'MPI-ESM1-2-HR'	SSP 585
	'MPI-ESM1-2-LR'	SSP 585
	'MRI-ESM2'	SSP 585
	'NorESM2-LM'	SSP 585
